# CXCL13 is a predictive biomarker in idiopathic multicentric Castleman disease

**DOI:** 10.1038/s41467-022-34873-7

**Published:** 2022-11-24

**Authors:** Sheila K. Pierson, Laura Katz, Reece Williams, Melanie Mumau, Michael Gonzalez, Stacy Guzman, Ayelet Rubenstein, Ana B. Oromendia, Philip Beineke, Alexander Fosså, Frits van Rhee, David C. Fajgenbaum

**Affiliations:** 1 Center for Cytokine Storm Treatment & Laboratory, Department of Medicine, University of Pennsylvania, Philadelphia, PA 19104 USA; 2 Medidata Solutions, New York, NY 10014 USA; 3 Department of Oncology, Oslo University Hospital, Oslo, Norway; 4 K.G. Jebsen Centre for B-cell Malignancies, University of Oslo, Oslo, Norway; Oslo University Hospital, Oslo, 0372 Norway; 5 Myeloma Center, University of Arkansas for Medical Sciences, Little Rock, AR 72205 USA

**Keywords:** Predictive markers, Acute inflammation, Chemokines, Proteomic analysis

## Abstract

Idiopathic multicentric Castleman disease (iMCD) is a rare and poorly-understood cytokine storm-driven inflammatory disorder. Interleukin-6 (IL-6) is a known disease driver in some patients, but anti-IL-6 therapy with siltuximab is not effective in all patients, and biomarkers indicating success at an early time point following treatment initiation are lacking. Here we show, by comparison of levels of 1,178 proteins in sera of healthy participants (*N* = 42), patients with iMCD (*N* = 88), and with related diseases (*N* = 60), a comprehensive landscape of candidate disease mediators and predictors of siltuximab response. C-X-C Motif Chemokine Ligand-13 (CXCL13) is identified and validated as the protein most prominently up-regulated in iMCD. Early and significant decrease in CXCL13 levels clearly distinguishes siltuximab responders from non-responders; a 17% reduction by day 8 following siltuximab therapy initiation is predictive of response at later time points. Our study thus suggests that CXCL13 is a predictive biomarker of response to siltuximab in iMCD.

## Abstract (web summary)

Idiopathic multicentric Castleman disease (iMCD) is a life-threatening inflammatory disease requiring immediate intervention, for which the recommended first-line therapy is the Interleukin-6 pathway inhibitor siltuximab. Authors here show that the change in levels of the chemokine CXCL13 shortly following the start of siltuximab treatment is predictive of response.

## Introduction

Human herpes virus 8 (HHV8)-negative/idiopathic multicentric Castleman (iMCD) disease (iMCD) is a heterogeneous inflammatory disorder with an unknown etiology^1^. It is characterized by extensive generalized lymphadenopathy with characteristic histopathology and cytokine-driven inflammation that may progress to life-threatening, multi-organ failure in severe cases. Within iMCD, there exist distinct clinical phenotypes. Patients who present with thrombocytopenia, anasarca, fever/elevated C reactive protein (CRP), reticulin fibrosis or renal failure, and organomegaly are defined as iMCD-TAFRO, while those who do not meet these criteria are referred to as iMCD not otherwise specified (iMCD-NOS). iMCD-NOS patients typically have a milder disease course, and some demonstrate thrombocytosis and hypergammaglobulinemia. Patients are also classified according to histopathologic subtype, including hyaline vascular/hypervascular, plasmacytic, or mixed, though the clinical implications of defining histopathologic subtype is unclear.

Interleukin-6 (IL-6), a pro-inflammatory cytokine, is a well-established disease driver in a portion of iMCD cases^2,3^. This discovery led to the development and use of anti-IL-6 therapies, tocilizumab—directed against the IL-6 receptor, and siltuximab—directed against IL-6^4,5^. While tocilizumab is approved for iMCD in Japan, siltuximab is the only drug approved for iMCD in the United States and Europe and is recommended as first-line therapy based on its effectiveness in approximately 34–50% of iMCD cases^6^. Patients who do not respond to siltuximab often need intensive chemotherapy and a brief window of time exists to make this treatment decision. However, it is difficult to predict which patients will respond to siltuximab and can take weeks to determine if it is having an effect. We recently identified and validated a 7-protein panel consisting of apolipoprotein E, amphiregulin, serum amyloid P-component, inactivated complement C3b, immunoglobulin E, IL-6, and erythropoietin that was able to predict a subset of patients with a significantly higher likelihood of response to siltuximab (65 vs 19%)^7^. The clinical utility of this panel is currently limited, given that these proteins are not routinely quantified in a clinical setting and the specific thresholds included in the algorithm need to be further refined. A single, simple biomarker that could be tested shortly after initiation of siltuximab and indicate whether a patient is likely to benefit from treatment would be preferable.

Few treatment options exist for patients who do not respond to siltuximab, and optimal second-line therapy is unknown. Siltuximab-refractory iMCD patients often receive combination chemotherapy, which is associated with significant toxicity and unclear efficacy. Pathways involving mTOR and JAK/STAT have been recently identified as candidate treatment targets^7–9^, but their effectiveness and utility are still unclear and notable side effects have been reported. It is not known what other pathways or soluble factors contribute to pathogenesis in iMCD that could potentially serve as therapeutic targets, particularly among siltuximab non-responders. A pilot plasma proteomics study previously identified cytokines and chemokines, including C-X-C Motif Chemokine Ligand 13 (CXCL13 or B lymphocyte chemoattractant (BLC)) that were upregulated during iMCD flare^10^. CXCL13 is integral to immune homeostasis, the adaptive immune response, and lymph node morphology, all of which are disrupted in iMCD. CXCL13, which is produced by a subset of lymph node stromal cells (follicular dendritic cells, FDC) and T follicular helper (Tfh) cells, is responsible for homing CXCR5 + B cells to lymph node germinal centers. Increased circulating levels of CXCL13 may indicate increased germinal center activity and/or immune dysregulation, but the clinical significance of these proteins in iMCD is unknown.

Herein, we perform proteomic quantification of 1178 serum analytes in iMCD, healthy donors, and comparator diseases to identify disease mediators and analyze longitudinal samples in a subset of iMCD patients (*N* = 73) to search for early indicators of response to siltuximab. We discover that CXCL13 is one of the most upregulated proteins in iMCD patient sera relative to healthy donors. We also identify and validate CXCL13 as an early indicator of response to siltuximab. We show that CXCL13 levels rapidly decline in siltuximab responders but not in non-responders. Our data suggest that serum CXCL13 levels can indicate whether a patient will respond to anti-IL-6 therapy and may also be a therapeutic target pending further investigation.

## Results

### Serum proteomics identifies CXCL13 as a top upregulated protein between iMCD and healthy controls

To identify potential disease mediators and therapeutic targets in iMCD, we quantified serum proteins in a cohort of 88 iMCD patients (Primary cohort) relative to healthy and disease controls using a multiplex DNA-aptamer-based assay (SomaLogic SOMAScan). Our discovery cohort of 88 iMCD patients included 73 patients who participated in phase II registrational trial for siltuximab (NCT01024036) and 15 additional patients experiencing active disease^5^. All patient samples were collected prior to receiving treatment with siltuximab.

**Fig. 1:**
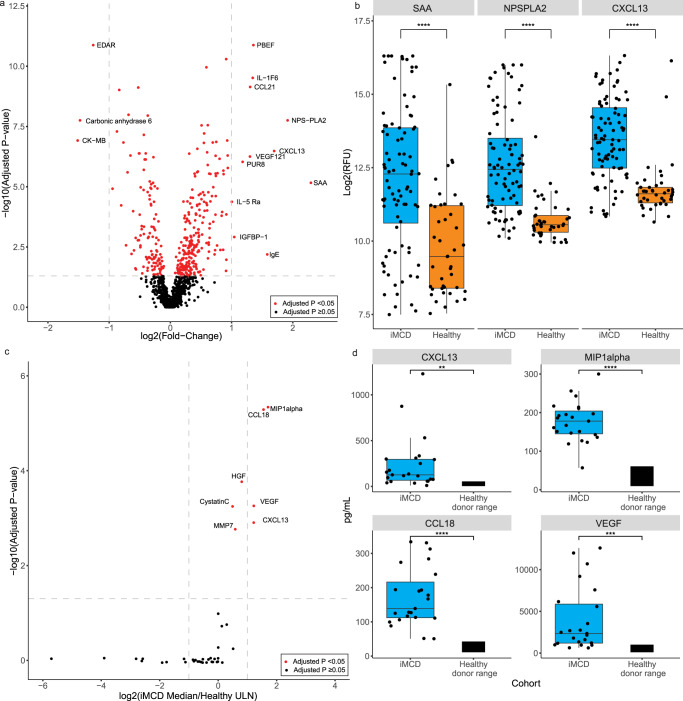
Differential expression of proteins between iMCD and healthy controls demonstrates increased CXCL13 across cohorts. **a** Volcano plot visualization of differentially expressed proteins between iMCD (*n* = 88) and healthy donors (*n* = 42). To detect differences in the proteome, linear regression models comparing iMCD to healthy, adjusted for age and sex, were run on each analyte. Results were adjusted by Benjamini & Hochberg method with alpha <0.05. We identified 251 upregulated proteins and 118 downregulated proteins in iMCD relative to healthy. Significant proteins that are >2-fold upregulated are labeled. Exact *P* values are listed in Supplementary Data 1 and the source data file. **b** The three most upregulated proteins (log2 transformed) in iMCD (*n* = 88) relative to healthy controls (*n* = 42) are visualized by boxplot. **c** To validate the proteomic changes identified against healthy individuals, we compared samples obtained from an independent validation iMCD cohort (*n* = 23) to the expected upper limit in a healthy population using an orthogonal platform. The median analyte level in iMCD was compared to the 97.5th percentile of the expected healthy range using a one-sided Mann–Whitney *U*-test with adjustment by Benjamini & Hochberg, alpha <0.05. A one-sided volcano plot of the 40 overlapping proteins quantified is shown. Proteins with median levels confirmed to be significantly elevated in iMCD over the 97.5th percentile of the expected healthy range are labeled. Exact *P* values are listed in Supplementary Table 1 and the source data file. **d** Box plots of the absolute concentrations (pg/mL) of the four proteins with the largest fold-change in iMCD (*n* = 23) compared to the healthy range are shown. The healthy donor range represents the 2.5th to 97.5th percentiles for healthy donors according to the RBM Human Discovery Map v1.0 platform. CCL18 C-C motif chemokine ligand 18, CCL21 C-C motif ligand 21, CK-MB creatine kinase-MB, CXCL13 C-X-C motif chemokine ligand 13, EDAR tumor necrosis factor receptor superfamily member EDAR, HGF hepatocyte growth factor, IgE immunoglobulin E, IGFBP-1 insulin-like growth factor-binding protein 1, IL-1F6 interleukin-36 alpha, IL-5 Ra interleukin-5 receptor subunit alpha, MIP1a macrophage inflammatory protein-1 alpha, MMP7 matrix metallopeptidase 7, NPS-PLA2 phospholipase A2, PBEF pre-B-cell colony-enhancing factor, PUR8 adenylosuccinate lyase, SAA serum amyloid A, VEGF vascular endothelial growth factor, ULN upper limits of normal. Box plots include a center line (median), box limits (upper and lower quartiles), whiskers (1.5x interquartile range), and all data points. *P < 0.05, **P < 0.01, ***P < 0.001, ****P < 0.0001. Source data are provided as a Source Data file.

We first performed a differential expression analysis between the 88 iMCD patients and 42 healthy controls (Fig. 1a and Supplementary Data 1). Of the 1178 analyzed proteins, 369 were found to be significantly up- or downregulated (all false discovery rate (FDR) <0.05) between iMCD and healthy controls, with 251 (68%) upregulated and 118 (32%) downregulated. Fourteen of these proteins demonstrated greater than twofold up- (*N* = 11) or down-regulation (*N* = 3) relative to healthy controls (Supplementary Data 1). These 14 proteins include acute phase reactants (phospholipase A2 (NPS-PLA2), serum amyloid A (SAA)), chemokines and cytokines (pre-B-cell colony-enhancing factor (PBEF), interleukin-36 alpha (IL-1F6), C-C Motif Chemokine Ligand 21 (CCL21), CXCL13, vascular endothelial growth factor (VEGF)), and cytokine receptors and binding proteins (tumor necrosis factor receptor superfamily member EDAR (EDAR), interleukin-5 receptor subunit alpha (IL-5 Ra), insulin-like growth factor-binding protein 1 (IGFBP-1)), among others (Carbonic anhydrase 6, creatine kinase-MB (CK-MB), adenylosuccinate lyase (PUR8), immunoglobulin E (IgE)). Notably, 8 of these 14 top differentially expressed proteins had previously been identified as greater than twofold up- or downregulated in iMCD flare relative to remission in a pilot proteomic study of six patients^10^ and the top three proteins were identical: NPS-PLA2, SAA, and CXCL13 (Fig. 1b).

To confirm the proteomic alterations we observed, we utilized an orthogonal multiplex immunoassay (RBM Human Discovery Map v 1.0) to compare the serum proteome of an independent cohort of 23 iMCD patient samples (Validation cohort) to expected healthy ranges. All 23 patients in the Validation cohort were enrolled in the siltuximab phase I dose-finding study *(*NCT00412321)^11^. Among the 190 quantified analytes, 40 were mapped to one of the 251 significantly upregulated proteins from our analysis. For each of these analytes, we compared the median (50th percentile) levels in iMCD to the 97.5th percentile levels in healthy donors to identify proteins significantly increased over healthy levels. Of the 40 proteins analyzed, there were seven proteins with a median value significantly greater than the 97.5th percentile of healthy individuals (FDR <0.05) (Fig. 1c and Supplementary Table 1). CXCL13 was identified among the seven and one of four that were twofold upregulated over the 97.5th percentile of healthy individuals, with the other three being macrophage inflammatory protein-1 alpha (MIP1a), C-C motif chemokine ligand 18 (CCL18), and VEGF (FDR <0.0001) (Fig. 1d).

### Serum CXCL13 elevated across iMCD clinical subtypes and differentially expressed relative to clinicopathologic overlapping disorders

**Fig. 2:**
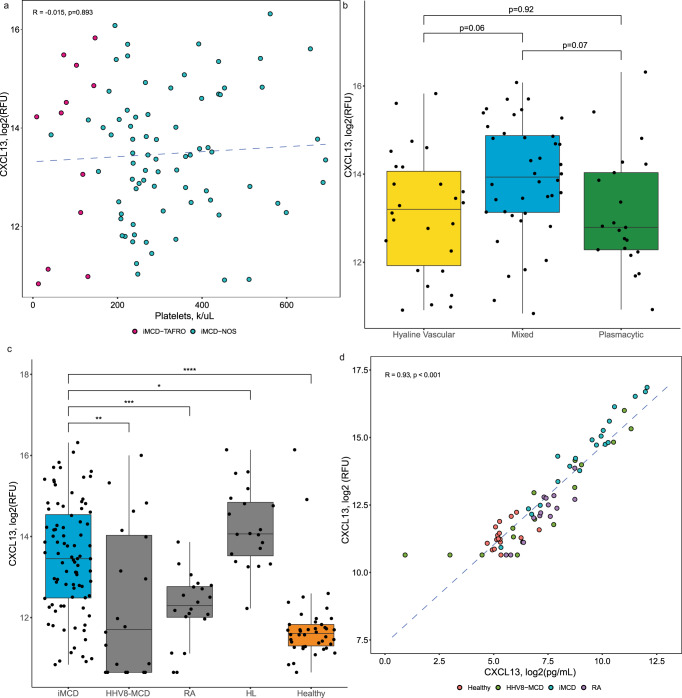
Serum CXCL13 differentially expressed in iMCD compared to clinicopathologically overlapping diseases. **a** Spearman rank correlation test of log2 CXCL13 against platelets in iMCD (*n* = 88) at the time of sample draw. There was no discernable relationship (*R* = −0.015, *p* = 0.89). **b** Box plots showing the log2 normalized relative fluorescent unit (RFU) concentrations of CXCL13 in iMCD patients with hyaline vascular (*n* = 26), mixed (*n* = 40), and plasmacytic (*n* = 21) histopathology. Analysis of variance followed by a two-tailed post hoc *t*-test with Bonferroni adjustment was used to test for differences according to histopathological subtype There was no difference between histopathological subtypes (hyaline vascular vs mixed histopathology, *p* = 0.06; plasmacytic vs hyaline vascular, *p* = 0.92; and plasmacytic vs mixed, *p* = 0.07. **c** Box plots showing the log2 normalized RFU concentrations of CXCL13 in iMCD compared to the related disorders and healthy donors. iMCD (*n* = 88) differed from each HHV8-MCD (*n* = 20), RA (*n* = 20), HL (*n* = 20), and healthy (*n* = 42). Statistical significance of disease status was determined with a linear regression model with age, sex, and CRP as covariates, and a two-tailed Wald test was used to test pairwise differences with iMCD, with multiple comparisons Bonferroni corrected (RA: *p* = 5.6 × 10^−4^; HL: *p* = 0.03; HHV8-MCD: *p* = 0.003; healthy: *p* = 3.3 × 10^−7^). **d** Pearson correlation test demonstrating a strong linear correlation between log2(CXCL13) as measured by the SomaSCAN multiplex DNA-aptamer assay and by an ELISA immunoassay (*n* = 66) *R* = 0.93; 95% confidence interval: 0.89–0.96, *p* = 1.7 × 10^−29^). Box plots include a center line (median), box limits (upper and lower quartiles), whiskers (1.5x interquartile range), and all data points. *P < 0.05, **P < 0.01, ***P < 0.001, ****P < 0.0001. Source data are provided as a Source Data file.

The identification of CXCL13 as one of the most significantly increased proteins in two independent iMCD cohorts relative to healthy controls was particularly noteworthy since we previously found CXCL13 to be the most upregulated cytokine between flare and remission in a pilot proteomic study^10^. Given the heterogeneity observed across iMCD clinical subtypes and histopathologic subtypes, we next investigated CXCL13 levels in the serum of iMCD patients in the Primary cohort according to inferred clinical phenotype (iMCD-TAFRO or iMCD-NOS) and histopathologic subtype (hyaline vascular, plasmacytic, mixed). Clinical phenotype was not systematically recorded in phase II clinical trial for siltuximab, from which 73 of the 88 iMCD patient samples were collected; therefore, confirmation of clinical phenotype was not possible. However, low platelet levels are highly indicative of iMCD-TAFRO^12^ and were used as a proxy to infer clinical phenotype. Patients with platelets <150 k/µL were defined as iMCD-TAFRO (*N* = 10), and the remaining patients were defined as iMCD-NOS (*N* = 78). Interestingly, we did not find a significant difference in CXCL13 levels between the clinical phenotypes (*t* = 0.07; *p* = 0.95; Supplementary Fig. 1). We also evaluated the correlation between serum CXCL13 and platelet levels and did not find a relationship (*R* = −0.06, *p* = 0.89; Fig. 2a). Lastly, we investigated serum CXCL13 according to histopathological subtype (*F* = 3.8; *p* = 0.03). After adjusting for multiple comparisons, we found no differences between subtypes (hyaline vascular (*N* = 26) vs. mixed (*N* = 40), *p* = 0.056; hyaline vascular vs. plasmacytic (*N* = 21), *p* = 0.92; mixed vs plasmacytic, *p* = 0.071). Of note, the implications of the histopathological subtype is difficult to interpret given the spectrum of histopathological features and inconsistency with classifying patients by histopathological subtype^13^.

To assess whether the upregulated CXCL13 observed in iMCD was a product of nonspecific inflammation or may point to a specific feature or mechanism in iMCD, we investigated CXCL13 levels in the serum relative to disorders with overlapping clinicopathologic features. We evaluated the differential expression of CXCL13 in the 88 iMCD patient samples in the Primary cohort compared to 20 rheumatoid arthritis (RA) patients, 20 Hodgkin lymphoma (HL) patients, and 20 HHV8-associated MCD (HHV8-MCD) patients. These diseases were selected for comparison because they represent diseases of autoimmune, neoplastic, and infectious origin, respectively. After adjusting for age, sex, and CRP (as a proxy for inflammation), we found a significant relationship between diagnosis and CXCL13 expression (*p* < 0.0001). iMCD patients demonstrated significantly higher CXCL13 expression in the serum compared to each RA (*p* = 5.6 × 10^−4^) and HHV8-MCD (*p* = 0.003). However, they demonstrated significantly lower CXCL13 expression compared to HL (*p* = 0.03; Fig. 2c). To validate the results found by the multiplex aptamer assay, which provides relative quantification, we quantified levels of CXCL13 in a subset of available serum samples from iMCD, RA, HHV8-MCD, and healthy donors in the Primary cohort by an enzyme-linked immunosorbent assay (ELISA) assay and found a strong correlation (*R* = 0.93, *p* < 0.001), supporting our results (Fig. 2d). Overall, our findings reveal that serum CXCL13 is elevated across both clinical phenotypes and histopathological subtypes compared to healthy donors. Lastly, CXCL13 levels are elevated compared to two related, non-malignant inflammatory conditions (RA, HHV8-MCD) but not HL, which has been found to involve the CXCR5/CXCL13 axis in pathogenesis. Further research is needed into a host of related inflammatory diseases to determine if serum CXCL13 could be used as a specific differentiator from at least a portion of related diseases.

### Increased lymph node expression of CXCL13 in iMCD-TAFRO compared to iMCD-NOS and reactive lymph nodes

**Fig. 3:**
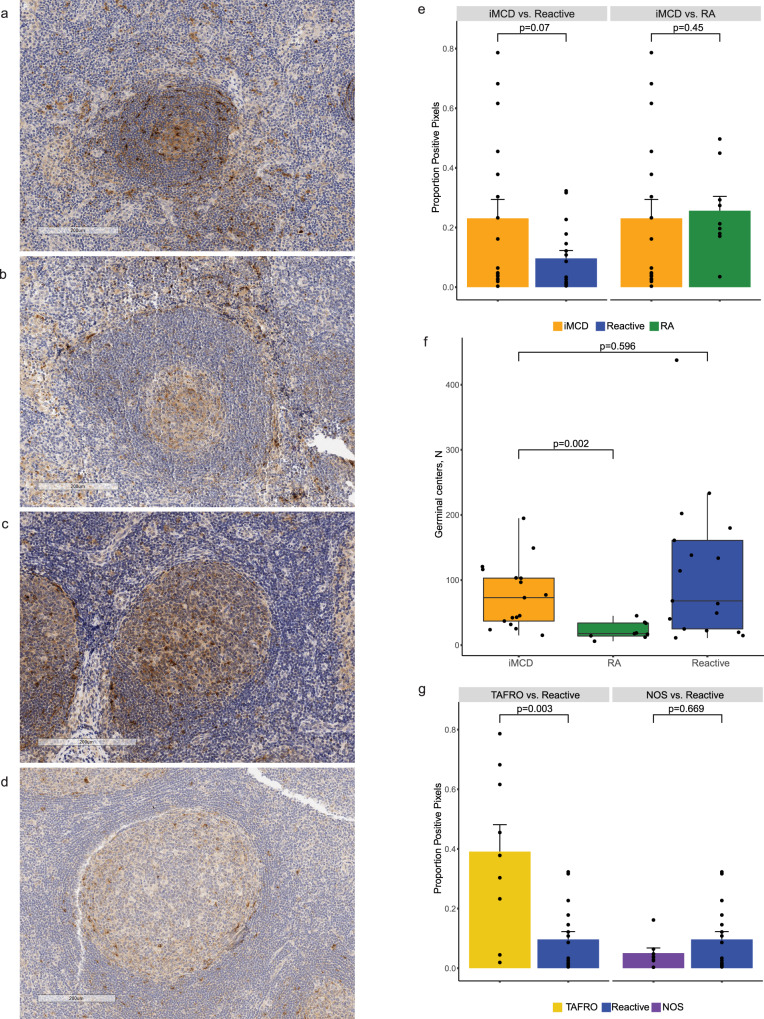
CXCL13 expression in the lymph node demonstrates a difference between iMCD-TAFRO and reactive but not iMCD-NOS and reactive. Representative germinal centers from **a** iMCD-TAFRO, **b** iMCD-NOS, **c** RA, and **d** reactive lymph nodes, bars represent 200 µm. **e** Comparison of positive pixel intensity in iMCD (*n* = 17) with reactive (*n* = 17; *p* = 0.07) and RA (*n* = 9; *p* = 0.45). The error bar represents the standard error of the mean. **f** Comparison of the number of germinal centers in iMCD (*n* = 17), RA (*n* = 9), and reactive (*n* = 17) lymph nodes. iMCD lymph nodes have significantly more germinal centers than RA (*p* = 0.002) but not reactive (*p* = 0.596). **g** Comparison of positive pixel intensity between iMCD-TAFRO (*n* = 9) and reactive (*n* = 17) and between iMCD-NOS (*n* = 8) and reactive. iMCD-TAFRO demonstrates significantly more CXCL13 expression than reactive (*p* = 0.003), but there is no difference between iMCD-NOS and reactive (*p* = 0.67). The error bar represents the standard error of the mean. Statistical differences were determined by a two-tailed non-parametric normal scores test. Box plots include a center line (median), box limits (upper and lower quartiles), whiskers (1.5x interquartile range), and all data points. Source data are provided as a Source Data file.

Next, we extended our investigation of CXCL13 to the lymph node given that CXCL13 is believed to be primarily produced in lymph node tissue, abnormal lymph node morphology is required to diagnose iMCD, and we previously showed increased expression of CXCL13 in iMCD lymph nodes compared to normal lymph node tissue^10^. We performed immunohistochemistry and evaluated CXCL13 expression in lymph nodes collected from 17 iMCD patients (Immunohistochemistry cohort), including nine patients who met validated iMCD-TAFRO criteria^12^ and eight patients who demonstrated consistency with iMCD-NOS, as well as in patients with reactive lymph nodes that demonstrated some iMCD-like features (*N* = 17) and RA lymph nodes (*N* = 9) (representative images Fig. 3a–d). Reactive lymph nodes, which were initially felt to be consistent with iMCD but later determined to not fulfill consensus diagnostic criteria upon expert pathology review, were selected as the primary comparison group because we wanted to assess whether CXCL13 expression in lymph node tissue was increased compared to patients with a related inflammatory syndrome and reactive lymph node changes. A specific and objective tissue-based biomarker to distinguish these iMCD-like cases from definitive iMCD cases would help with diagnosis and treatment triage. RA was also selected as a positive control comparator disease given its clinicopathological overlap with iMCD and its established link between circulating CXCL13 levels and disease activity^14,15^.

When comparing all iMCD lymph node samples to reactive lymph node germinal centers, there was a marginal but statistically insignificant increase in positive staining in germinal centers of iMCD (*t* = −1.8; *p* = 0.07) and there was no difference between iMCD and RA (*t* = −0.8; *p* = 0.45) (Fig. 3e). We next examined the number of germinal centers per lymph node area for each group and found that iMCD lymph nodes had significantly more germinal centers (mean (standard deviation, SD): 76.3 (50.4)) than RA (mean (SD): 22.2 (12.8); *t* = −3.1; *p* = 0.002), but no difference with reactive (mean (SD): 113.0 (110.0); *t* = 0.53; *p* = 0.596; Fig. 3f). The similar CXCL13 expression in germinal centers between iMCD and RA but increased serum CXCL13 in iMCD may be due to the increased germinal centers in each lymph node section and the fact that iMCD patients have far more enlarged lymph nodes than RA.

Given the wide range in staining and the phenotypic heterogeneity in iMCD, we investigated differences between each iMCD clinical phenotype and reactive lymph nodes. Interestingly, we found significantly higher CXCL13 staining in iMCD-TAFRO lymph nodes compared to reactive (*t* = −2.9; *p* = 0.003) but no difference between iMCD-NOS and reactive lymph nodes (*t* = 0.4; *p* = 0.669; Fig. 3g). This difference may reflect differences in histopathology as iMCD-TAFRO patients typically have more regressed germinal centers with fewer B cells whereas both iMCD-NOS and reactive lymph nodes often demonstrate hyperplastic germinal centers with many B cells.

While we did not confirm cell types expressing CXCL13, the staining pattern of the positively staining cells in the iMCD lymph nodes was most consistent with a diffuse, mesh-like pattern resembling stromal cells, though there were occasional round cells resembling lymphoid cells. These results extend our prior investigation of CXCL13 expression in the lymph node and suggest that CXCL13 expression patterns and cellular sources may differ between iMCD subtypes.

### CXCL13 demonstrates an early and significant decline in siltuximab responders compared to non-responders

After demonstrating increased CXCL13 in iMCD serum relative to healthy controls and differential levels relative to related disorders, we next set out to explore whether there are serum proteins that might be predictive of treatment response. To do so, we analyzed pretreatment and longitudinal samples collected from the 73 patients in the Primary cohort who participated in the phase II trial. Samples were collected on day 1 (immediately before treatment was administered) and on day 8 and day 64 of treatment.

First, we searched for biomarkers that demonstrated a significant difference between pretreatment samples from patients who responded to siltuximab (*N* = 17) and those who did not (*N* = 32) according to the phase II trial criteria. Interestingly, after adjusting for age, sex, and disease severity, there were no proteins significantly different between siltuximab responders and non-responders prior to treatment commencement (Supplementary Data 2). Immunoglobulin E (IgE), SAA, and MHC class I polypeptide-related sequence A (MICA) were the only three proteins with greater than the twofold difference between responders and non-responders, none of which achieved significance.

**Fig. 4:**
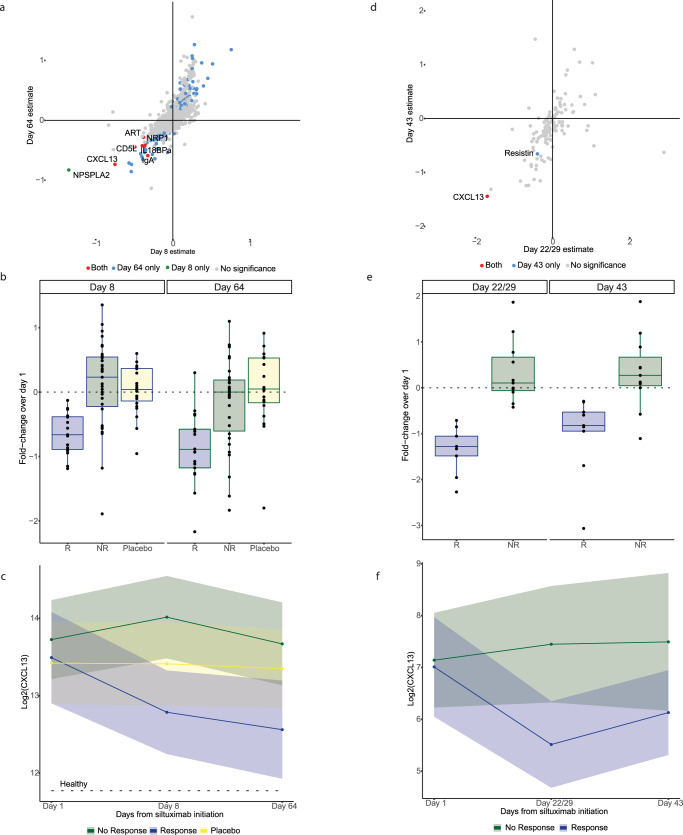
CXCL13 identified and validated as a potential early predictor of siltuximab response. **a** For each protein, a model was fit to assess the significance of the interaction of time point and response in patients in the phase II siltuximab trial; coefficient estimates at day 8 siltuximab treatment are plotted against those at day 64 of treatment and colored by significance. Eight proteins were significantly decreased in siltuximab responders compared to non-responders at both time points, including the labeled proteins CXCL13, immunoglobulin A (IgA), agouti-related protein (ART), CD5 antigen-like (CD5L), neuropilin-1 (NRP1), interleukin-18-binding protein (IL18Bpa), and two unlabeled proteins, tumor necrosis factor receptor superfamily member 17 (BCMA) and beta-2-microglobulin (b2M). NPS-PLA2 was significantly decreased in responders on day 8 but was not significant by day 64. **b** Log2 fold-change and **c** mean (95% confidence interval) of CXCL13 at day 8 and day 64 in responders (*n* = 17), non-responders (*n* = 32), and placebo (*n* = 24) patients of the siltuximab phase II trial. **d** Coefficient estimates of overlapping proteins quantified in an independent cohort (phase I siltuximab trial) at day 22/29 of siltuximab treatment plotted against those at day 43 and colored by significance. CXCL13 is the only protein identified in both cohorts that was significant at both time points. Resistin was significant at day 43 only. **e** Log2 fold-change and **f** mean (95% confidence interval) of CXCL13 at day 22/29 and day 43 in responders (*n* = 10) and non-responders (*n* = 13) in the phase I siltuximab trial. Box plots include a center line (median), box limits (upper and lower quartiles), whiskers (1.5x interquartile range), and all data points. Source data are provided as a Source Data file.

With no pretreatment predictive biomarkers identified, we explored whether changes in serum protein levels shortly after administration of siltuximab were associated with response and could potentially guide treatment decisions. To identify these changes, we analyzed the available longitudinal samples. Using a linear mixed-effects model adjusted for disease activity, age, sex, and the time point–sex interaction, differences between responders and non-responders were identified by the significance of time point (day 8 and day 64) and response. On treatment day 8, 9 proteins were significantly different in responders compared to non-responders (FDR <0.05), including immunoglobulin A (IgA), Tumor necrosis factor receptor superfamily member 17 (BCMA), NPS-PLA2, agouti-related protein (ART), interleukin-18-binding protein (IL-18 BPa), CD5 antigen-like (CD5L), beta-2-microglobulin (b2M), CXCL13, and neuropilin-1 (NRP1) (Fig. 4a). All nine of these proteins were significantly decreased in responders compared to non-responders. At day 64, the number of proteins with a significant interaction between response and time point increased to 121 (FDR <0.05), including eight of the nine proteins from day 8; NPS-PLA2 was the only protein significant at day 8 that did not remain significant at day 64. CXCL13 showed a strikingly consistent reduction in responders compared with non-responders and placebo-treated patients. While CXCL13 levels of responders approached the levels of healthy donors, CXCL13 levels in non-responders and placebo-treated patients remained elevated relative to healthy donor levels (Fig. 4b, c). Notably, there was no difference in the level of CXCL13 levels between responders and non-responders at baseline (FDR = 0.83). These results indicate that there may be serum indicators of response as early as day 8 and that, among 1178 analytes quantified, CXCL13 serum quantification is a noteworthy discovery and may be a candidate biomarker to indicate siltuximab response or non-response in iMCD patients.

### Early significant decline in CXCL13 validated in siltuximab responders

To validate the finding that there is early differential protein levels associated with siltuximab response, we compared differences in protein levels over time between siltuximab responders (*N* = 10) and non-responders (*N* = 13) in 23 iMCD patients (Validation cohort). From this cohort, samples were collected on day 1 (pretreatment), day 22 or 29 (day 22/29), and day 43 and quantified using the multiplex immunoassay. We tested our model on the overlapping proteins measured in the independent cohort. Interestingly, at day 22/29, we found the difference in CXCL13 to be significant (FDR = 0.002) but no other differences among the other 132 proteins. At day 43, there were two proteins that demonstrated significant differences between responders and non-responders: CXCL13 (FDR = 0.009) and resistin (FDR = 0.005) (Fig. 4d). Despite the smaller cohort, different sample collection time points, and different proteomic quantification platforms, CXCL13 was again identified as a potential biomarker in iMCD (Fig. 4e, f). Notably, CXCL13 was the only protein that was significantly different between responders and non-responders at the respective early (day 8 and day 22, respectively) and late (day 64 and day 43, respectively) time points in both the Primary and Validation cohorts. These results suggest that CXCL13 could be used as an early indicator of response to siltuximab.

### A serum decrease in CXCL13 of ≥17% after siltuximab administration is predictive of response

**Fig. 5:**
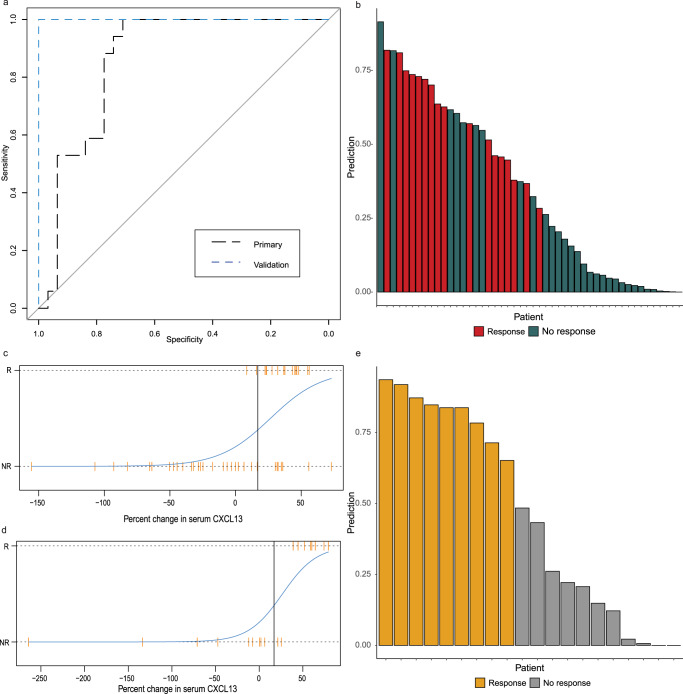
Decreased serum CXCL13 early in siltuximab treatment is predictive of future response. **a** Receiver operating characteristic (ROC) curve for the prediction of siltuximab response based on serum levels of CXCL13 in the training cohort (*n* = 48; 31 non-responders, 17 responders) (AUC = 0.86 [CI:0.753–0.966])) and the test cohort (*n* = 20; 11 non-responders, 9 responders) (AUC = 1.0) **b** Waterfall plot of response prediction values by patient colored by true response status in the training cohort. **c** Classification by logistic regression in the training cohort. Responders plotted along the top horizontal bar and non-responders along the bottom horizontal bar. The x-axis represents the percent change in CXCL13 from the baseline. The logistic regression curve is plotted in blue, with a vertical line drawn at a 17% reduction. By day 8, a 17% reduction in CXCL13 has an 82% recall, 82% true positive rate, 23% false positive rate, 79% accuracy, and 67% precision in response prediction in the training cohort. **d** Classification by logistic regression in the test cohort. By day 22/29, 17% reduction in CXCL13 has a 100% recall, 100% true positive rate, 18% false positive rate, 90% accuracy, and 92% precision, and **e** waterfall plot of response prediction values by patient colored by true response status in the test cohort. Source data are provided as a Source Data file.

Given that changes in CXCL13 occur differentially in siltuximab responders and non-responders, we sought to determine the optimal threshold for minimum percent change in CXCL13 serum levels that would predict response to siltuximab. We already showed that there is a significant relationship between response and CXCL13 at day 8, so we anticipated that pretreatment change in CXCL13 would likewise yield significant results; however, identifying the minimum percent reduction in CXCL13 that is predictive of response is of meaningful clinical use. We found that a 17% reduction in CXCL13 serum levels by day 8 of treatment in the Primary cohort had the optimal sensitivity (0.82) and specificity (0.77), with an accuracy of 79.2% and precision of 66.7% (area under the curve, (AUC) = 0.86 (0.75–0.97; *p* = 0.002) (Fig. 5a–c). We applied the model to the Validation cohort and obtained an AUC of 1.0, indicating that the cohort had perfect separation, with all responders having at least a 17% reduction in CXCL13 by day 22/29, and two non-responders having at least a 17% reduction by day 22/29 (accuracy 90%, precision 81.8%) (Fig. 5a, d, e). Our results are suggestive that a decrease in CXCL13 of 17% or greater after initiation of siltuximab indicates that a patient is likely to respond to siltuximab and that CXCL13 quantification in the clinic could help to guide continued use of siltuximab or initiation of additional therapies if sufficient CXCL13 reduction is not achieved.

## Discussion

Interrogation of the serum proteome in a large group of iMCD patients demonstrated that CXCL13 is an early indicator of response to siltuximab and is a top upregulated protein in iMCD. These discoveries address one of the most pressing challenges for iMCD: timely treatment decision-making. Though strictly correlative, the significant upregulation across iMCD and the rapid reduction in CXCL13 in iMCD patients who respond to siltuximab but not in patients who do not respond suggest that CXCL13 may be a critical component to pathogenesis and thus a therapeutic target warranting further investigation.

While a previous pilot study (*N* = 6) using the same multiplex DNA-aptamer platform (SomaLogic SOMAscan) had identified CXCL13 as the top upregulated cytokine in serum during iMCD flare relative to remission^10^, the patient sample size in this study is larger, represents a broader spectrum of disease severity, and is compared to a set of healthy donors and related diseases. Further, our results are validated on samples from the same cohort using an ELISA and on a separate cohort by an orthogonal proteomic assay. Despite differences between groups and platforms, CXCL13 was the most upregulated cytokine in serum compared to healthy individuals and was differentially expressed compared with RA, HL, and HHV8-MCD. Interestingly, though the average level of CXCL13 was significantly increased in iMCD compared to healthy donors, RA, and HHV8-MCD, it was significantly decreased compared to HL, which has been recently found to demonstrate increased CXCL13-CXCR5 signaling^16^. Further diseases will need to be studied to determine if serum CXCL13 levels could be used clinically to differentiate iMCD from at least some clinicopathologically overlapping diseases.

Based on our previous finding of increased CXCL13 expression in germinal centers from iMCD patients versus normal lymph node tissue, we investigated lymph node expression of CXCL13. Tissue-based analysis of CXCL13 revealed a marginal but non-significant increased expression per unit area in germinal centers relative to reactive lymph nodes and no difference compared to RA lymph nodes. Given the small sample size and the fact that all of the reactive lymph nodes were previously misdiagnosed as iMCD by at least one pathologist, there may have been clearer differences if a larger cohort or a cohort of reactive lymph nodes without iMCD-like features were used. Further, CXCL13 levels in the serum and joints of RA patients have been found to be an established biomarker in RA^14^; therefore, RA would be expected to have increased CXCL13 and the lack of a difference between iMCD and RA does not contradict a potential role for CXCL13 in iMCD. Though these results did not demonstrate that germinal centers from iMCD patients express more CXCL13 per unit area than germinal centers of RA or reactive lymph nodes, we did find more germinal centers per lymph node section in iMCD than RA and iMCD patients have substantially more enlarged lymph nodes than patients with reactive lymph nodes and RA patients, which could explain the increased circulating levels^17^. The staining pattern in the iMCD lymph nodes most closely resembled a stromal cell pattern, which likely represents FDCs, though there were occasional lymphoid-appearing cells, which likely represent Tfh cells. Tfh cells have been shown to express increased levels of CXCL13 in diseases like angioimmunoblastic T cell lymphoma (AITL)^18^; dysregulations of Tfh cells may likewise contribute to iMCD pathogenesis^19^. Notably, there was a significant difference in CXCL13 expression between iMCD-TAFRO and reactive but no difference between iMCD-NOS and reactive. Given that we found no difference in serum levels of CXCL13 between patients presumed to be iMCD-TAFRO compared to iMCD-NOS, this may be due to different numbers of germinal centers per lymph node or numbers of enlarged lymph nodes. Alternatively, it may suggest different cellular sources of circulating CXCL13 between subtypes. iMCD-TAFRO germinal centers are regressed with fewer B cells; therefore, Tfh cells and FDCs may be overexpressing CXCL13 to home more B cells to the germinal centers. In comparison, iMCD-NOS patients often have hyperplastic germinal centers with increased B cells, so there may be less germinal center-derived CXCL13 to home B cells and more extra-follicular CXCL13 expression from compartments such as the bone marrow or circulating immune cells.

Given that these results implicate excess CXCL13, possibly as a result of dysregulated FDCs or TFh cells, in iMCD pathogenesis, the early and significant decline of CXCL13 in siltuximab-responders versus non-responders was highly notable. We have previously searched for predictive biomarkers of response. We identified a model of clinical parameters, including hemoglobin, immunoglobulin G, fibrinogen, and CRP, which discriminated responders from patients who failed treatment^20^, but this has never been validated, and interpretation of the model coefficients are limited. Additionally, our recently published 7-protein panel is able to differentiate a subgroup of patients with a higher likelihood of response to siltuximab but it is not generalizable to the clinic at this time^7^. This study identified a single predictive biomarker. Prior to treatment, CXCL13 was significantly higher in this cohort of iMCD patients than in age-matched healthy donors. By day 64, CXCL13 levels in siltuximab responders decreased to levels approaching the healthy donor range but remained elevated in non-responders and placebo patients. Remarkably, these results were validated in a separate cohort that included a smaller patient subset and different protein quantification assay. These data suggest that quantification of CXCL13 pretreatment as early as day 8 may represent an early predictive biomarker for an eventual response to IL-6-directed treatment. This does not, however, imply that IL-6 blockade should be immediately abandoned based on an early determination of CXCL13 levels. In the randomized trial, a number of patients benefited from siltuximab clinically despite failing to reach a formal response as per protocol criteria. The addition of other treatments, such as chemotherapy, early in the treatment course may be a more conservative approach. Therefore, given the critical nature of iMCD, patients who do not achieve a 17% reduction following treatment administration may still need to remain on siltuximab and have additional treatments added.

The upregulation of CXCL13 and the correlation between changes in CXCL13 and improvement in disease activity is striking and suggests that CXCL13 could also be a treatment target in iMCD. An antibody targeting CXCL13–mediated signaling has demonstrated efficacy in mouse models of autoimmune diseases^21^ and could be considered for a clinical trial in iMCD. Additional treatment targets in iMCD have recently been identified^8,22,23^, but preliminary data suggest varying effects and unclear safety profiles, so investigation of additional therapeutic targets like CXCL13 is warranted in this vulnerable patient population.

There are several limitations to this study. First, combining all iMCD cases together may be a limitation given that we’ve previously shown that iMCD is proteomically heterogeneous^7^. To address this, we performed sub-analyses of CXCL13 levels in likely iMCD-TAFRO versus iMCD-NOS patients in serum and lymph node samples. While definitive identification of iMCD-TAFRO was not possible in the Primary cohort, we did not identify differences in CXCL13 between the likely iMCD-TAFRO and like iMCD-NOS groups. We also looked for a correlation with low platelet counts, which is strongly associated with iMCD-TAFRO and did not find one. Additionally, we looked at associations with CXCL13 levels between histopathologic subtypes and found no differences between histopathological subtypes after adjustment. Histopathology is difficult to interpret given the inconsistency with assigning subtypes and the general lack of clinical implications of histopathologic subtype^13^. Second, there is a lower proportion of patients meeting the criteria for the iMCD-TAFRO clinical subtype in this study compared to what we see in clinical practice^12^. However, we did actively seek out iMCD-TAFRO samples from collaborators to be included in this study along with the largely non-TAFRO cohort from the siltuximab clinical trials. Further, the 17 patients in our immunohistochemistry analysis included 9 iMCD-TAFRO patients. Third, RA, HL, and HHV8-MCD were selected as representative autoimmune, neoplastic, and infectious diseases with clinicopathologically overlapping features to iMCD, but other diseases may have been better suited for direct comparison in a diagnostic setting. Other cytokine storm disorders and reactive inflammatory conditions should be compared with iMCD in the future^24^. Similarly, RA was selected as a positive control for CXCL13 tissue staining because of tissue availability and prior work demonstrating increased CXCL13 in RA patients^14^, but another disease such as AITL would have also been suitable. Fourth, significant work is still needed to advance these insights into clinical laboratory improvement amendments (CLIA)-certified tests that are clinically actionable. For instance, the timing of measuring CXCL13 after initiation of siltuximab needs to be optimized such that there is sufficient time for changes in CXCL13 levels while keeping the test within a clinically meaningful window. Finally, additional mechanistic work is needed to determine if CXCL13 is also a therapeutic target.

Our study highlights the role of CXCL13 as a predictive biomarker of response to first-line treatment in iMCD. Further exploration of CXCL13 performance in clinical settings to inform treatment decisions as well as its role as a therapeutic target is warranted.

## Methods

### Patient characteristics and consent

Previously banked biosamples from three independent cohorts were analyzed herein, including a cohort of patients whose serum was quantified by multiplex DNA-aptamer-based technology (Primary cohort), a cohort of patients whose serum was quantified by multiplex assay (Validation cohort), and a cohort of patients whose lymph node tissue was studied (Immunohistochemistry cohort). All patients in this study provided informed consent for samples to be used in research, and all uses of human materials included herein was approved by the Quorum Review Institutional Review Board and the University of Pennsylvania Institutional Review Board. The reporting of clinical data herein complies with STROBE guidelines. Patients did not receive compensation for their participation in this study. Demographics for all three cohorts and a study flow can be found in Supplementary Table 2 and Supplementary Fig. 2, respectively.

### Inclusion and ethics statement

The researchers acknowledge the importance of collaboration with researchers in local settings. This research did not include an investigation in resource-poor areas or specific settings.

#### Primary cohort

iMCD patients recruited for the Primary cohort include 79 patients who participated in phase II registrational trial for siltuximab (NCT01024036)^5^ and 19 additional patients experiencing active disease—a total of 98 iMCD patients. The 79 patients in the trial were randomized to placebo (*N* = 26) or to siltuximab (*N* = 53). Siltuximab response was determined by durable symptomatic and tumor (radiologic lymph node) response criteria. Samples were collected from all iMCD patients before siltuximab treatment. Additionally, longitudinal samples at day 8 and day 64 of siltuximab treatment were collected from the iMCD patients in the phase II trial. Infusions took place after sample collection on day 1 (pretreatment), day 22, day 43, and day 64. Samples from 88 patients achieved quality thresholds and were included in the analysis.

One-time samples were collected from comparator groups, including 44 healthy donors, recruited to serve as age- and sex-matched comparators to the iMCD group (42 met quality thresholds), and 20 patients each with clinicopathologically overlapping disorders RA, HL, and HIV-positive, HHV8-MCD—a form of MCD that is caused by uncontrolled infection with HHV8—representing diseases of autoimmune, neoplastic, and infectious origin respectively.

#### Validation cohort

Patients recruited for the Validation cohort include 25 iMCD patients who were enrolled in the siltuximab phase I dose-finding study *(*NCT00412321)^11^. Siltuximab was infused every 2 to 3 weeks in this dose-finding study. Pretreatment samples (day 1) and samples collected on day 22 or 29 (day 22/29) and day 43 of siltuximab treatment were collected from all patients in cohort 2. Siltuximab response was determined by radiologic lymph node response criteria^11^. Samples from 23 patients achieved quality thresholds and were included in the analysis.

Disease activity in both cohorts was calculated using CRP, hemoglobin, and albumin according to published guidelines^7,25^.

#### Immunohistochemistry cohort

Patients recruited for the Immunohistochemistry cohort include 19 iMCD patients, including 11 iMCD-TAFRO and 8 iMCD-NOS 11 RA patients, and 18 patients with reactive lymph nodes that were considered to demonstrate iMCD-like changes by at least one pathological review. Stored specimens from iMCD and reactive lymph node tissue were obtained from the ACCELERATE Natural History registry (NCT02817997)^26^ and Castleman biobank; RA lymph nodes were collected from the University of Pennsylvania biobank. Samples from 17 iMCD patients, 9 RA patients, and 17 patients with reactive lymph nodes met tissue and staining quality and were included in the analysis.

### Relative proteomic quantification by multiplex DNA-aptamer

Quantification of 1305 protein analytes in the Primary cohort was performed by SomaLogic SOMAscan 1.3k version^27^, a DNA-based aptamer technology per the manufacturer’s protocol. This study was done in accordance with SomaLogic standard operating procedures and best practices. Specifically, all samples were shipped to SomaLogic on dry ice to be run at their central facility. Due to the number of samples, quantification was performed in two batches, and five technical replicates were included between the two batches. Samples were transferred at random to a plate, therefore healthy controls and iMCD samples, as well as other cohorts, were randomized across plates. Calibrators and quality control samples are interspersed throughout the plate at random during the transfer. The calibrators were used in the plate scaling and calibration process to reduce batch variance. SomaLogic SOMAscan pre-processing was performed, and 1178 analytes passed quality control. Data were log2 transformed and capped at 2.5th and 97.5th percentiles^7^. Batches were bridged together by the average log ratio of technical replicates for each analyte. Following quality control sample exclusion, pretreatment samples available for analysis included 88 iMCD patients, 42 healthy donors, and 20 each of RA, HL, and HHV8-MCD. Longitudinal samples available for analysis included samples from day 8 and day 64 of treatment from 73 iMCD patients.

### Absolute proteomic quantification by multiplex immunoassay

Concentrations of 190 proteins were quantified in the Validation cohort using the RBM Human Discovery Map v 1.0 platform^28^. RBM provided data for the healthy control 2.5th and 97.5th percentiles, representing the low and high ends of the normal range, which was reportedly based on approximately 100 healthy individuals. Values were standardized and log2 transformed. Levels of proteins denoted as having a concentration below the detection limit were imputed with the least detectable dose, defined as the concentration interpolated from the mean, plus 3 standard deviations, of 20 standard diluent blank readings, a marker of the assay limit of sensitivity. As applicable, proteins measured by RBM were mapped to targets in the SomaLogic platform by UniProt ID. A listing of all possible mapped targets can be found in Supplementary Data 3.

### Quantification of CXCL13 by enzyme-linked immunosorbent assay (ELISA)

CXCL13 was additionally quantified by enzyme-linked immunoassay (ELISA) in a subset of samples that had undergone protein quantification by SomaLogic SOMAScan. In total, 69 samples had sufficient volume remaining for quantification by ELISA, including 20 iMCD, 15 HHV8-MCD, 14 RA, and 20 healthy. The levels of CXCL13 were measured using the Human CXCL13 Quantikine kit (R&D Systems) following the manufacturer’s instructions. Absorbance measurements were read using an Emax microplate reader (Molecular Devices) and associated software, SoftMax Pro v5.4.6 (Molecular Devices).

### Immunohistochemistry

CXCL13 expression in lymph nodes of the Immunohistochemistry cohort was determined by immunohistochemistry staining of formalin-fixed paraffin-embedded (FFPE) lymph node tissue. Staining was performed by the Pathology Core at the Children’s Hospital of Philadelphia. Slides were generated at 5-micron thickness. Epitope retrieval was done for 20 min with an E2 retrieval solution (Leica Biosystems). Immunohistochemistry was performed on a Leica Bond Max automated staining system (Leica Biosystems) using the Bond Intense R staining kit (Leica Biosystems DS9263). Polyclonal rabbit anti-CXCL13 (AF801, R&D Systems) was used at a 1:500 dilution and an extended incubation time of 1 h at room temperature. Avidin-Biotin Blocking was added (Vector Labs SP- 2001) and a Peptide Blocking step was included (DAKO X0909). Slides were digitally scanned at 20x magnification on an Aperio ScanScope CS-O slide scanner (Leica Biosystems).

Blinded reviewers performed lymph node germinal center annotation and analysis offline using Aperio ImageScope v 12.4.0.5043 and Image Analysis Toolkit software (color deconvolution v9 algorithm). Five slides were excluded due to poor tissue or staining quality. The strength of pixel intensity was estimated according to the color deconvolution algorithm, and thresholds for none, weak, medium, and strong pixel intensity were applied to all lymph nodes^10^. The percentage of areas meeting medium and strong staining was recorded as achieving a positive pixel threshold for differential expression analysis.

### Statistical analysis

To detect differences in the iMCD proteome relative to healthy individuals, linear models comparing iMCD prior to treatment with siltuximab with healthy individuals with age and sex covariates were run on each analyte. False discovery rate (FDR) was determined using Benjamini & Hochberg method with alpha <0.05^29^. Validation of proteomic changes identified against healthy individuals was performed using a one-sided Mann–Whitney *U*-test against 97.5th normal percentiles, as defined above, for each selected analyte. A one-sided test was selected given the hypothesis that the levels of the identified analytes would be greater at the 50th percentile in iMCD than at the 97.5th percentile in healthy controls.

To test for a difference in serum CXCL13 between iMCD-TAFRO and iMCD-NOS prior to siltuximab treatment, we performed a two-sample *t*-test. Spearman’s rank was used to test the correlation between platelets and serum CXCL13. Analysis of variance followed by post hoc *t*-test with Bonferroni adjustment was used to evaluate CXCL13 according to histopathological subtype. To determine the differential expression of serum CXCL13 between iMCD and related disorders (HL, RA, and HHV8-MCD), we regressed disease status and clinical covariates (age, sex, CRP) on CXCL13. A likelihood ratio test was used to evaluate the overall significance of the disease, and the Wald test with Bonferroni adjustment was used to test pairwise differences with iMCD. Pearson correlation coefficient was used to determine the correlation between CXCL13 measured by multiplex DNA-aptamer and CXCL13 measured by ELISA.

To test for a difference in positive-threshold CXCL13 staining expression and the number of germinal centers in the lymph nodes of iMCD patients and selected comparator groups and between iMCD-TAFRO and iMCD-NOS we performed a two-sided normal score test (Van der Waerden test), a non-parametric method by which data were converted to ranks and then standard normal distribution quantiles^30^.

Baseline differences between siltuximab responders and non-responders were detected using Benjamini & Hochberg corrected linear models adjusted for age, sex, and disease severity. To detect whether kinetic changes in serum proteomics levels were associated with response to siltuximab, linear mixed-effects models using a random intercept were fit to each protein. Timepoint was treated as a categorical variable with three values—pretreatment (day 1), day 8, and day 64. Pretreatment was treated as the reference. Differences between responders and non-responders were assessed by the interaction between time point and response, and baseline activity score, age, sex, and the interaction of sex and time point were included as covariates. The patient was included as the random effect. Validation of results was performed by fitting the linear mixed model to each overlapping analyte quantified by RBM in the 23 independent iMCD patients from the phase I trial. All results were FDR adjusted at 0.05 by Benjamini & Hochberg.

Lastly, logistic regression was performed to determine the optimal threshold for minimum percent change in CXCL13 from pretreatment to day 8 that indicates the response to siltuximab indicative of response to siltuximab. A receiver operating characteristic (ROC) curve was fit to the Primary dataset, composed of patients from the phase II siltuximab trial, and to the Validation dataset, composed of patients from the phase I siltuximab trial. The predictive threshold of response was determined by identifying the probability that corresponded to the point on the ROC that simultaneously maximized sensitivity and specificity. Classification metrics were computed according to the selected threshold.

Statistical analysis and figure generation was performed using R v4.0.5. All data cleaning and analysis was performed through publicly available R packages: linear modeling was performed with lme4 v1.1-26 and lmerTest v3.1-3, normal scores test was performed with snpar v1.0; receiver operating characteristic curve was generated using ROCR v1.0-1.1 and pROC v1.18.0. Other packages required for data cleaning and figure generation include: dplyr v1.0.5, ggplot2 v3.3.5, smplot v0.1.0, reshape2 v1.4.4, stringr v1.4.0, readxl v1.3.1.

### Reporting summary

Further information on research design is available in the Nature Portfolio Reporting Summary linked to this article.

## Supplementary information

Supplementary Information

Description of Additional Supplementary Files

Supplementary Data 1

Supplementary Data 2

Supplementary Data 3

Reporting Summary

## Data Availability

The proteomic and immunohistochemistry data generated in this study have been deposited in the Zenodo Repository under accession code [10.5281/zenodo.7010937] and at the Gene Expression Omnibus (GEO) Repository under accession code GSE217351. UniProt database (https://www.uniprot.org/) was used to link proteins quantified on both SomaLogic SOMAScan v1.3k and RBM Human Discovery Map v1.0. Source data are provided with this paper.

## Source data

Source Data
