# Supplementary material for: CXCL13 is a predictive biomarker in idiopathic multicentric Castleman disease
Source: Nat Commun. 2022 Nov 24;13:7236. doi: 10.1038/s41467-022-34873-7 (PMC9700691; doi:10.1038/s41467-022-34873-7)
Supplement: Supplementary file 1 — Supplementary Information [file 41467_2022_34873_MOESM1_ESM.pdf]

Supplementary Information

CXCL13 is a predictive biomarker in idiopathic multicentric Castleman disease

Sheila K. Pierson[1*], Laura Katz[2], Reece Williams[1], Melanie Mumau[1], Michael Gonzalez[1], Stacy Guzman[1], Ayelet Rubenstein[1], Ana B. Oromendia[2], Philip Beineke[2], Alexander Fosså[3,4], Frits van Rhee[5], David C. Fajgenbaum[1*]

[1]Center for Cytokine Storm Treatment & Laboratory, Department of Medicine, University of Pennsylvania, Philadelphia, PA, 19104, USA

[2]Medidata Solutions, New York, NY, 10014, USA

[3]Department of Oncology, Oslo University Hospital, Oslo, Norway

[4]K.G. Jebsen Centre for B-cell Malignancies, University of Oslo, Oslo, Norway; Oslo University Hospital, Oslo, 0372, Norway

[5]Myeloma Center, University of Arkansas for Medical Sciences, Little Rock, AR, 72205, USA

*Co-Corresponding Authors:

Supplementary Fig. 1. Serum CXCL13 demonstrates no difference between iMCD-TAFRO and iMCD-NOS patients.

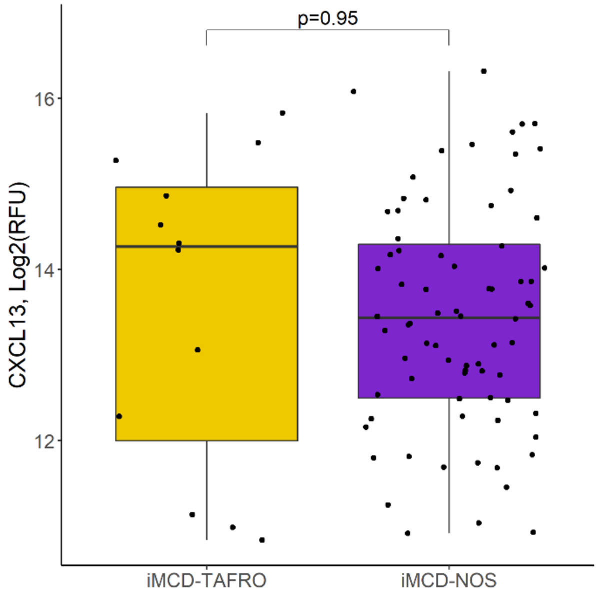

Box plot showing the log2 normalized relative fluorescent unit concentration of CXCL13 in patients identified as likely iMCD-TAFRO (n=10; platelets <150 k/µL) compared to iMCD-NOS (n=78; platelets ≥150 k/µL), p=0.95. Statistical difference evaluated by two-tailed t-test. Box plots include a center line (median), box limits (upper and lower quartiles), whiskers (1.5x interquartile range), and all data points.

Supplementary Fig. 2. Flowchart demonstrating our cohort design and sample selection approach.

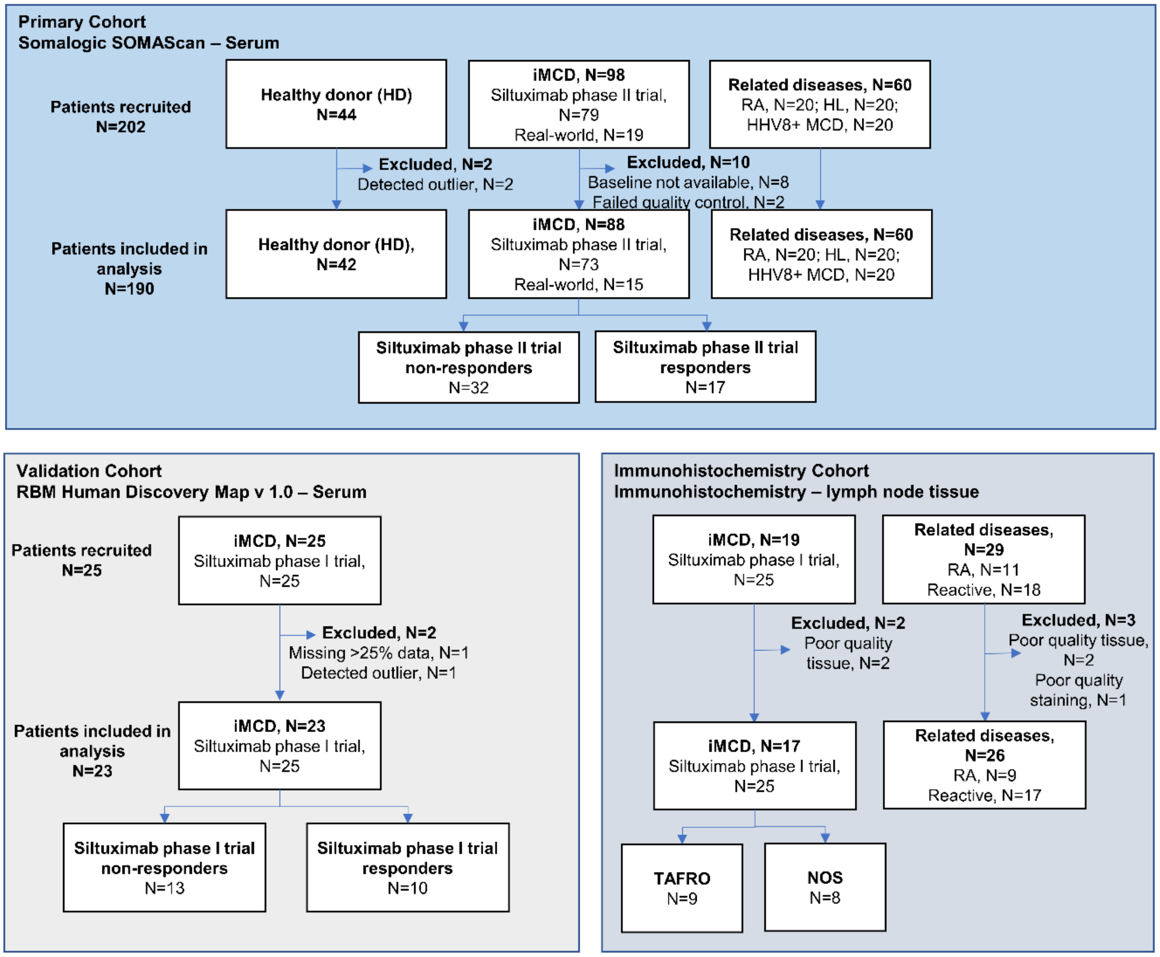

Serum samples from iMCD, healthy donor, and comparator disease samples were analyzed through the Somalogic SOMAScan. Results were validated on an independent cohort through an orthogonal platform, RBM Human Discovery Map v 1.0. Immunohistochemistry staining of CXCL13 in lymph node tissue was performed in a third cohort.

Supplementary Table 1. Comparison of iMCD median analyte levels to upper limits of normal for a healthy population

Target	Abbreviation	Log2 Fold-change	Unadjusted p-value	Adjusted p-value
C-C Motif Chemokine Ligand 18 (CCL18)	CCL18	1.71	1.19E-07	4.77E-06
Macrophage Inflammatory Protein-1 alpha (MIP-1 alpha)	MIP-1 alpha	1.55	2.38E-07	4.77E-06
Vascular Endothelial Growth Factor (VEGF)	VEGF	1.22	6.39E-05	0.001
C-X-C Motif Chemokine Ligand-13 (CXCL13)	CXCL13	1.21	2.04E-04	0.001
Hepatocyte Growth Factor (HGF)	HGF	0.81	1.44E-05	1.92E-04
Matrix Metalloproteinase-7 (MMP-7)	MMP-7	0.58	3.19E-04	0.002
Tenascin-C (TN-C)	TN-C	0.51	0.147	0.521
Cystatin-C	Cystatin-C	0.49	5.96E-05	0.001
Vascular Cell Adhesion Molecule-1 (VCAM-1)	VCAM-1	0.29	0.046	0.184
Placenta Growth Factor (PLGF)	PLGF	0.14	0.398	1.000
FASLG Receptor (FAS)	FAS	0.12	0.046	0.184
Nerve Growth Factor beta (NGF-beta)	NGF-beta	0.00	0.018	0.092
Erythropoietin (EPO)	EPO	0.00	0.156	0.521
Interleukin-2 (IL-2)	IL-2	0.00	0.993	1.000
Prolactin (PRL)	PRL	-0.01	0.422	1.000
Complement C3 (C3)	C3	-0.02	0.363	1.000
Insulin-like Growth Factor-Binding Protein 2 (IGFBP-2)	IGFBP-2	-0.04	0.759	1.000
Matrix Metalloproteinase-1 (MMP-1)	MMP-1	-0.12	0.857	1.000
Agouti-Related Protein (AGRP)	AGRP	-0.18	0.994	1.000
Tumor Necrosis Factor Receptor-Like 2 (TNFR2)	TNFR2	-0.22	0.982	1.000
Resistin	Resistin	-0.26	0.668	1.000
Bone Morphogenetic Protein 6 (BMP-6)	BMP-6	-0.33	0.996	1.000
Alpha-1-Antichymotrypsin (AACT)	AACT	-0.40	1.000	1.000
E-Selectin	E-Selectin	-0.52	0.991	1.000
Osteopontin	Osteopontin	-0.53	0.996	1.000
Beta-2-Microglobulin (B2M)	B2M	-0.58	0.999	1.000
Thrombopoietin	Thrombopoietin	-0.64	1.000	1.000
CD5 (CD5L)	CD5L	-0.70	0.993	1.000
Immunoglobulin M (IGM)	IGM	-0.78	0.981	1.000
Chemokine CC-4 (HCC-4)	HCC-4	-0.83	0.998	1.000
Myeloid Progenitor Inhibitory Factor 1 (MPIF-1)	MPIF-1	-0.86	1.000	1.000
Angiopoietin-2 (ANG-2)	ANG-2	-1.12	0.999	1.000

Intercellular Adhesion Molecule 1 (ICAM-1)	ICAM-1	-1.17	1.000	1.000
Tumor Necrosis Factor alpha (TNF-alpha)	TNF-alpha	-1.77	1.000	1.000
Macrophage Migration Inhibitory Factor (MIF)	MIF	-1.92	1.000	1.000
Interleukin-25 (IL-25)	IL-25	-2.58	1.000	1.000
Pregnancy-Associated Plasma Protein A (PAPP-A)	PAPP-A	-2.61	1.000	1.000
Interleukin-6 (IL-6)	IL-6	-2.81	1.000	1.000
Tissue Factor (TF)	TF	-3.90	1.000	1.000
Immunoglobulin E (IgE)	IgE	-5.71	1.000	1.000

To validate the proteomic changes identified against healthy individuals, we compared samples obtained from an independent validation iMCD cohort (n=23) to the expected upper limit in a healthy population using an orthogonal platform. The median analyte level in iMCD was compared to the 97.5th percentile of the expected healthy range using a one-sided Mann-Whitney U test with adjustment by Benjamini & Hochberg, alpha <0.05.

Supplementary Table 2. Demographics and disease characteristics of iMCD patients in each analysis cohort

	Primary Cohort N=88	Validation Cohort N=23	IHC cohort N=17
**Age**			
Mean (SD)	51.8 (13.7)	51.3 (12.1)	36.9 (17.4)
Range	24, 85	24, 76	2, 66
**Sex**			
Female	28 (31.8)	12 (52.2)	7 (41.2)
Male	60 (68.2)	11 (47.8)	10 (58.8)
**Race**			
Asian	40 (45.5)	2 (8.7)	3 (17.6)
Black	4 (4.5)	2 (8.7)	2 (11.8)
Hawaiian or Pacific Islander	2 (2.3)	0	0
Native American	2 (2.3)	0	1 (5.9)
White	35 (39.8)	19 (82.6)	10 (58.8)
Other	2 (2.3)	0	0
Unknown	2 (2.3)	0	1 (5.9)
**Clinical subtype**			
TAFRO	10 (11.4)	0	9 (52.9)
NOS	78 (88.6)	0	8 (47.1)
Unknown	0	23 (100)	0
**Histopathological subtype**			
Hyaline vascular	26 (29.5)	0	10 (58.8)
Mixed	40 (45.5)	0	4 (23.5)
Plasmacytic	21 (23.9)	0	3 (17.6)
Not reported	1 (1.1)	23 (100)	0
**Clinical Trial**			
Siltuximab phase I	0	23	0
Responder	0	10 (43.5)	0
Non-responder	0	13 (56.5)	0
Siltuximab phase II	73	0	0
Placebo Arm	24 (32.9)	0	0
Siltuximab Arm	49 (67.1)	0	0
Responder	17 (34.7)	0	0
Non-responder	32 (65.3)	0	0
Unknown	0	0	17 (100)
**C reactive protein, mg/L**			
Mean (SD)	42.1 (52.1)	40.0 (43.9)	113 (82.3)
**Hemoglobin, g/dL**			
Mean (SD)	11.8 (2.54)	11.9 (2.31)	8.6 (1.53)
**Albumin, g/dL**			
Mean (SD)	3.44 (0.71)	3.5 (0.74)	2.7 (0.51)
**Platelets, k/uL**			
Mean (SD)	298 (156)	343 (260)	274 (242)

Abbreviations: IHC, Immunohistochemistry; SD, standard deviation; IQR, interquartile range; TAFRO, thrombocytopenia, anasarca, fever/elevated C reactive protein, renal failure/reticulin fibrosis, organomegaly; NOS, not-otherwise-specified.
