# Supplementary material for: CXCL13 is a predictive biomarker in idiopathic multicentric Castleman disease
Source: Nat Commun. 2022 Nov 24;13:7236. doi: 10.1038/s41467-022-34873-7 (PMC9700691; doi:10.1038/s41467-022-34873-7)
Supplement: Supplementary file 2 — Description of Additional Supplementary Files [file 41467_2022_34873_MOESM2_ESM.pdf]

Description of Additional Supplementary Files

File Name: Supplementary Data 1
Description: Proteomic differential expression between iMCD and healthy donors. To detect differences in the iMCD proteome relative to healthy individuals, linear models comparing iMCD (n=88) prior to treatment with siltuximab with healthy individuals (n=42) with age and sex covariates were run on each analyte. False discovery rate (FDR) was determined using Benjamini & Hochberg method with alpha <0.05.

File Name: Supplementary Data 2
Description: Proteomic differential expression at baseline (pretreatment) between siltuximab responders (n=17) and siltuximab non-responders (n=32). Baseline differences between siltuximab responders (n=17) and non-responders (n=32) were detected using linear models adjusted for age, sex, and disease severity. Benjamini & Hochberg method was used to correct for multiple comparisons with alpha <0.05.

File Name: Supplementary Data 3
Description: Mapped targets between SomaLogic SOMAScan and RBM Human Discovery Map v 1.0 (n=133)
