# Supplementary material for: CXCL13 is a predictive biomarker in idiopathic multicentric Castleman disease
Source: Nat Commun. 2022 Nov 24;13:7236. doi: 10.1038/s41467-022-34873-7 (PMC9700691; doi:10.1038/s41467-022-34873-7)
Supplement: Supplementary file 6 — Reporting Summary [file 41467_2022_34873_MOESM6_ESM.pdf]

nature portfolio

Corresponding author(s): Sheila K. Pierson
David C. Fajgenbaum

Last updated by author(s): Nov 3, 2022

Reporting Summary

Nature Portfolio wishes to improve the reproducibility of the work that we publish. This form provides structure for consistency and transparency in reporting. For further information on Nature Portfolio policies, see our Editorial Policies and the Editorial Policy Checklist.

Statistics

For all statistical analyses, confirm that the following items are present in the figure legend, table legend, main text, or Methods section.

n/a	Confirmed	
☐	☒	The exact sample size (*n*) for each experimental group/condition, given as a discrete number and unit of measurement
☐	☒	A statement on whether measurements were taken from distinct samples or whether the same sample was measured repeatedly
☐	☒	The statistical test(s) used AND whether they are one- or two-sided<br>*Only common tests should be described solely by name; describe more complex techniques in the Methods section.*
☐	☒	A description of all covariates tested
☐	☒	A description of any assumptions or corrections, such as tests of normality and adjustment for multiple comparisons
☐	☒	A full description of the statistical parameters including central tendency (e.g. means) or other basic estimates (e.g. regression coefficient) AND variation (e.g. standard deviation) or associated estimates of uncertainty (e.g. confidence intervals)
☐	☒	For null hypothesis testing, the test statistic (e.g. *F*, *t*, *r*) with confidence intervals, effect sizes, degrees of freedom and *P* value noted<br>*Give P values as exact values whenever suitable.*
☒	☐	For Bayesian analysis, information on the choice of priors and Markov chain Monte Carlo settings
☐	☒	For hierarchical and complex designs, identification of the appropriate level for tests and full reporting of outcomes
☐	☒	Estimates of effect sizes (e.g. Cohen's *d*, Pearson's *r*), indicating how they were calculated

Our web collection on statistics for biologists contains articles on many of the points above.

Software and code

Policy information about availability of computer code

Data collection

Quantification of 1,305 protein analytes in the Primary cohort was performed by SomaLogic SOMAscan (1.3k version), a DNA-based aptamer technology per manufacturer's protocol. CXCL13 was additionally quantified by enzyme-linked immunoassay (ELISA) in a subset of samples that had undergone protein quantification by SomaLogic SOMAScan. Absorbance measurements were read using an Emax microplate reader (Molecular Devices) and associated software, SoftMax Pro v5.4.6 (Molecular Devices).

Concentrations of 190 proteins were quantified in the Validation cohort using the RBM Human Discovery Map v 1.0 platform.

Immunohistochemistry was performed on a Leica Bond Max automated staining system (Leica Biosystems) using the Bond Intense R staining kit (Leica Biosystems DS9263). Slides were digitally scanned at 20x magnification on an Aperio ScanScope CS-O slide scanner (Leica Biosystems).

Data analysis

Statistical analysis and figure generation was performed using R v4.0.5. Analysis was performed through previously published and publicly available R packages: linear modeling was performed with lme4 v1.1-26 and lmerTest v3.1-3, normal scores test was performed with snpar v1.0; receiver operating characteristic curve was generated using ROCR v1.0-1.1 and pROC v1.18.0. Other packages required for data cleaning and figure generation include: dplyr v1.0.5, ggplot2 v3.3.5, smplot v0.1.0, reshape2 v1.4.4, stringr v1.4.0, readxl v1.3.1

Immunohistochemistry analysis was performed using Apero ImageScope v 12.4.0.5043 and Image Analysis Toolkit software (color deconvolution v9 algorithm).

This manuscript does not involve the use of custom code.

For manuscripts utilizing custom algorithms or software that are central to the research but not yet described in published literature, software must be made available to editors and reviewers. We strongly encourage code deposition in a community repository (e.g. GitHub). See the Nature Portfolio guidelines for submitting code & software for further information.

Data

Policy information about availability of data

All manuscripts must include a data availability statement. This statement should provide the following information, where applicable:

- Accession codes, unique identifiers, or web links for publicly available datasets
- A description of any restrictions on data availability
- For clinical datasets or third party data, please ensure that the statement adheres to our policy

The proteomic and immunohistochemistry data generated in this study have been deposited in the Zenodo Repository under accession code 10.5281/zenodo.7010937 [https://doi.org/10.5281/zenodo.7010937] and at the Gene Expression Omnibus (GEO) Repositoryunder accession code GSE217351 [https://www.ncbi.nlm.nih.gov/geo/query/acc.cgi?acc=GSE217351]. UniProt database (https://www.uniprot.org/) was used to link proteins quantified on both SomaLogic SOMAScan v1.3k and RBM Human Discovery Map v1.0. Source data are provided with this paper.

Human research participants

Policy information about studies involving human research participants and Sex and Gender in Research.

Reporting on sex and gender	Sex of participants was determined based on self-report. Sex was included as a covariate in analyses. Gender was not collected.
Population characteristics	Samples were collected from a population of patients with idiopathic multicentric Castleman disease (iMCD).<br>Primary Cohort: The majority of iMCD patient samples collected came from patients in the phase II clinical trial (NCT01024036), whose characteristics have been published. Additional iMCD samples were collected from patients with a more severe phenotype in order to represent patients across the iMCD spectrum. Population characteristics are described in Table S3. Related disease samples were collected from patients with diseases that have overlapping characteristics, including HHV8+MCD, hodgkin lymphoma, and rheumatoid arthritis.<br>Validation Cohort: All iMCD patient samples collected came from patients in the phase I clinical trial (NCT00412321), whose characteristics have been published. Population characteristics are described in Table S3.<br>IHC Cohort: All iMCD patient samples and samples from patients with reactive lymph nodes came from patients enrolled in the ACCELERATE Natural History Registry and represent patients who have been diagnosed with iMCD by at least one pathologist. Population characteristics are described in Table S3.
Recruitment	Primary Cohort: The primary cohort resulted from samples collected from 98 iMCD patients, 20 rheumatoid arthritis (RA) patients, 20 Hodgkin lymphoma (HL) patients, 20 HHV8-associated multicentric Castleman disease (HHV8+MCD) patients, and 44 healthy donors. Of the 98 iMCD patients, 79 were initially recruited for the phase II clinical trial for siltuximab (NCT01024036) and provided longitudinal samples with consent for use in future studies. These patients were recruited into the clinical trial from 38 hospitals in 19 countries. An additional 19 patient iMCD samples, as well as the 60 related disease samples (RA, HL, and HHV8+MCD) and 44 healthy donor samples were collected from sample banks housed by collaborators located in Japan, Norway, the United Kingdom and 3 sites (University of Arkansas for Medical Sciences, Brigham and Women's Hospital, and University of Pennsylvania) in the United States. Healthy donor samples were collected from individuals with no known disorders who were roughly age and sex matched to the iMCD patient population. Healthy donors were primarily recruited from faculty and staff at the collaborating institutions who self-reported having no known disorders. Self-selection bias for healthy donors may be present ('healthy donor effect'). Specifically, individuals who opted in as a healthy donor volunteer may be more likely to have lower rates of morbidity rates and more active lifestyle compared to the general population (e.g. active in the workforce). It is possible that this may bias the comparison of iMCD patients to a general healthy population.<br><br>Validation Cohort: The validation cohort resulted from 25 iMCD patient samples collected from the phase I siltuximab trial (NCT00412321). The phase I trial recruited patients with Castleman disease, multiple myeloma, and non-Hodgkin lymphoma from 9 centers in the United States. Of the 67 patients treated in the trial, 37 patients were diagnosed with Castleman disease, and 25 of them had iMCD and provided a sample for proteomics research.<br><br>Immunohistochemistry Cohort: The immunohistochemistry cohort consisted of iMCD patient lymph node samples collected from patients enrolled in the ACCELERATE Natural History Registry (NCT02817997). Patients are recruited into ACCELERATE through the Castleman Disease Collaborative Network and physician outreach. Patients self-enroll into ACCELERATE, which may result in self-selection bias and is likely to include a population of patients with more severe disease. A panel of clinicians and pathologists review each case and confirm the accuracy of iMCD diagnosis.
Ethics oversight	Quorum Review Institutional Review Board, University of Pennsylvania Institutional Review Board

Note that full information on the approval of the study protocol must also be provided in the manuscript.

Field-specific reporting

Please select the one below that is the best fit for your research. If you are not sure, read the appropriate sections before making your selection.

- [x] Life sciences
- [ ] Behavioural & social sciences
- [ ] Ecological, evolutionary & environmental sciences

For a reference copy of the document with all sections, see nature.com/documents/nr-reporting-summary-flat.pdf

Life sciences study design

All studies must disclose on these points even when the disclosure is negative.

Sample size

Sample size calculation was not performed as no given hypothesis was tested. The intention of this study was to perform biomarker discovery and to search for alternative therapeutic targets. To that end, we assembled the largest feasible cohort of iMCD patient samples then available. To do so, we partnered with Janssen Pharmaceuticals to obtain longitudinal patient samples collected from 79 patients in the phase II siltuximab trial (NCT01024036), which was a large enough sample size to demonstrate differences between the siltuximab and placebo arms based on intention to treat and estimated effect size. To enhance this sample size for the present study, we sought additional samples from collaborators around the world. The total number of real-world iMCD samples collected (N=19) was based on available banked patient samples for this rare disease. At the time, no accessible biobank existed and available biosamples were limited given the rarity of the disease and wide geographic distribution of patients. Related disease sample sizes were based on feasibility of collecting samples with associated clinical data. Healthy control sample size was based on consultation with the SomaLogic proteomic discovery assay developers, with the recommendation to collect approximately 1 healthy for every 5 disease state samples. Additional healthy donors were sought until assay initiation.

To validate our findings from the primary cohort, we assembled two additional independent cohorts. The validation cohort included 25 iMCD patients who participated in the phase I siltuximab trial (NCT00412321) and provided longitudinal biosamples for proteomics research. This was the largest known independent validation cohort with available longitudinal samples systematically collected pre- and post-treatment with siltuximab.

Lastly, to investigate CXCL13 in the lymph node, lymph node samples were collected from 19 iMCD patients, 11 RA patients, and 18 reactive lymph nodes. iMCD and reactive lymph node sample sizes were based on 80% power and 5% two-sided level of significance to detect the effect size observed in a pilot study of 6 iMCD and 5 sentinal lymph nodes (Pierson et al., Am J Hematol, 2018). 11 RA samples were collected as a positive control and was based on availability from the sample bank.

Data exclusions

Outlier detection methods were established prior to analysis in order to identify samples that diverge from the overall population. Outliers were detected by aggregating the results of three conventional methods. Outliers were flagged if identified by 2 or more methods and were recommended for removal. Two samples from the primary cohort and from the validation cohort were found to be outliers based on at least 2 of these 3 methods: principal component analysis reconstruction residual, average pairwise distance (APW), and the APW to the K-nearest neighbors. These were excluded from further analysis.

Replication

Results obtained with the primary cohort were validated with an independent cohort of samples (validation cohort) obtained from the phase I siltuximab clinical trial (NCT00412321). Additionally, to validate the results found by the multiplex aptamer assay, which provides relative quantification, we quantified levels of CXCL13 in a subset of available serum samples from iMCD, RA, HHV8-MCD, and healthy donors in the Primary cohort by an enzyme-linked immunosorbent assay (ELISA) assay and found a strong correlation results. Validation of results are reported in the manuscript.

Randomization

This study involved proteomic quantification of previously collected and banked biospecimens and therefore randomization is not applicable to the present analysis.

A large proportion of biosamples collected for the primary cohort in this study were collected during the phase II clinical trial for siltuximab (NCT01024036). As part of that study design, patients were block randomized (block size 6) with a computer generated randomisation schedule in a ratio of 2 treatment to 1 control.

Blinding

This study involved proteomic quantification of previously collected and banked biospecimens and therefore blinding is not applicable to the present analysis. Technicians performing the proteomic quantification were however masked to sample identification.

This study includes a secondary analysis of samples collected as part of the phase II trial. As part of that study design, patients and investigators giving treatment were masked to allocation until protocol-defined failure, and investigators and independent assessors who evaluated outcomes were masked to allocation.

Reporting for specific materials, systems and methods

We require information from authors about some types of materials, experimental systems and methods used in many studies. Here, indicate whether each material, system or method listed is relevant to your study. If you are not sure if a list item applies to your research, read the appropriate section before selecting a response.

Materials & experimental systems

n/a	Involved in the study
	☒ Antibodies
☒	Eukaryotic cell lines
☒	Palaeontology and archaeology
☒	Animals and other organisms
	☒ Clinical data
☒	Dual use research of concern

Methods

n/a	Involved in the study
☒	ChIP-seq
☒	Flow cytometry
☒	MRI-based neuroimaging

Antibodies

Antibodies used

Polyclonal rabbit anti-CXCL13 (AF801, R&D Systems) was used at a 1:500 dilution

Validation

anti-CXCL13 antibody has been validated using an assay to assess its ability to neutralize CXCL13/BLC/BCA-1-induced chemotaxis in the BaF3 mouse pro-B cell line transfected with human CXCR5. The Neutralization Dose (ND50) is typically 1-4 µg/mL in the presence of 50 ng/mL Recombinant Human CXCL13/BLC/BCA-1. There are 14 citations on the manufacturer's website using this antibody.

Levels of CXCL13 were measured by ELISA using the Human CXCL13 Quantikine kit (R&D Systems) following manufacturer's instructions. Absorbance measurements were read using an Emax microplate reader (Molecular Devices) and associated software, SoftMax Pro v5.4.6 (Molecular Devices).

Clinical data

Policy information about clinical studies

All manuscripts should comply with the ICMJEguidelines for publication of clinical research and a completedCONSORT checklist must be included with all submissions.

Clinical trial registration

This study does not report the results of a clinical trial. However, patient samples were collected from the phase I study for siltuximab (NCT00412321), the phase II clinical trial for siltuximab (NCT01024036) and the phase IV natural history registry for patients with Castleman disease, ACCELERATE (NCT02817997).

Study protocol

Study protocols for the phase I and phase II siltuximab clinical trials can be found with their primary publications: van Rhee, F. et al. Siltuximab, a novel anti-interleukin-6 monoclonal antibody, for Castleman's disease. J. Clin. Oncol. 28, 3701–3708 (2010). and van Rhee, F. et al. Siltuximab for multicentric Castleman's disease: a randomised, double-blind, placebo-controlled trial. Lancet. Oncol. 15, 966–74 (2014).

Data collection

Primary Cohort: biospecimens were collected for this study were obtained during the phase II siltuximab trial (NCT01024036) conducted from 2010 to 2014. Specifically, patients were enrolled in 38 centers from 19 countries (China, United States, Brazil, Hong Kong, France, South Korea, Taiwan, Belgium, Russian Federation, Singapore, Israel, United Kingdom, New Zealand, Norway, Spain, Australia, Canada, Egypt, and Germany); the first patient was enrolled in February 2010 and the last patient's last visit occurred in February 2013. Additional real-world samples were collected from banked samples at sites in the United States (Philadelphia, Pennsylvania; Little Rock, Arkansas; and Boston, Massachusetts), the United Kingdom, Norway, and Japan. The additional real-world iMCD samples were colledcted and banked between April 2008 and July 2016. Rheumatoid arthritis samples were collected and banked between May 2003 and May 2015. Hodgkin lymphoma samples were collected and banked between November 2009 and September 2012. HHV8+MCD samples were collected and banked between April 2008 and November 2011. Healthy donor samples were collected and banked between November 2012 and July 2016.

Validation Cohort: biospecimens were collected during the phase I siltuximab trial (NCT00412321) conducted at 9 study centers in the United States. The first patient was enrolled in June 2005, and the last patient visit was April 2011.

IHC Cohort: tissue samples were collected from previously conducted clinical procedures to obtain diagnostic tissue. Patients were enrolled into the ACCELERATE natural history registry between October 2016 and May 2021, and diagnostic tissue was obtained thereafter.

Outcomes

This study does not report the results of a clinical trial. However, deidentified patient samples were collected from the phase II NCT01024036 and phase I NCT00412321 siltuximab studies for the purposes of performing proteomic quantification and searching for disease mediators and early indicators of response.

Phase II (NCT01024036): As reported in their primary publication, "the primary endpoint was durable tumour and symptomatic response for at least 18 weeks for the intention-to-treat population.... Tumour response was assessed by investigators and independent radiological review, masked to treatment failure (Biocor, Princeton, NJ, USA). Symptomatic response was assessed by investigators based on the sum of the severity of 34 disease-related signs and symptoms (disease related overall symptom score; appendix). Secondary endpoints were duration of tumour and symptomatic response, tumour response, time to treatment failure, 15 g/L or greater increase of haemoglobin concentration between baseline and week 13, dis continuation of corticosteroids, treatment failure rate, improvement of multicentric Castleman's disease-related symptoms, overall survival at 1 year, and patient-reported outcomes including changes from baseline in Functional Assessment of Chronic Illness Therapy—Fatigue score, Short Form-36 Health Survey subscale scores (data not shown), and Multicentric Castleman's Disease Signs and Symptom Scores." The results of these studies and additional information can be found in: van Rhee, F. et al. Siltuximab for multicentric Castleman's disease: a randomised, double-blind, placebo-controlled trial. Lancet. Oncol. 15, 966–74 (2014).

Phase I (NCT00412321): As reported in their primary publication, "The main efficacy end point of clinical benefit response (CBR) was defined as a composite of clinical and laboratory measures relevant to the management of CD. In addition, radiologic response was independently assessed by using modified Cheson criteria...CBR was defined as improvement from baseline (with no worsening in the remaining measures) in at least one of the following: >/= 2 g/dL increase in hemoglobin without transfusions; >/= 1 grade decrease in fatigue according to the National Cancer Institute Common Terminology Criteria of Adverse Events (NCI CTCAE), version 3.0; >/= 1 grade decrease in anorexia by NCI CTCAE; >/=2°C decrease in fever or return to 37°C or improvement in night sweats; >/= 5% increase in weight; or >/= 25% decrease bidimensionally in size of the largest lymph node." The results of this studies and additional information can be found in their primary publication: van Rhee, F. et al. Siltuximab, a novel anti-interleukin-6 monoclonal antibody, for Castleman's disease. J. Clin. Oncol. 28, 3701–3708 (2010).
